# Generalized anxiety disorder and online intelligence: A phenomenological account of why worrying is unhelpful

**DOI:** 10.1186/1747-5341-6-7

**Published:** 2011-05-03

**Authors:** Gerben Meynen

**Affiliations:** 1 Faculty of Philosophy, VU University Amsterdam, EMGO Institute, VU Medical Center Amsterdam, De Boelelaan 1105, 1081HV Amsterdam, The Netherlands

## Abstract

Worrying is the central feature of generalized anxiety disorder (GAD). Many people worry from time to time, but in GAD the worrying is prolonged and difficult to control. Worrying is a specific way of coping with perceived threats and feared situations. Meanwhile, it is not considered to be a helpful coping strategy, and the phenomenological account developed in this paper aims to show why. It builds on several phenomenological notions and in particular on Michael Wheeler's application of these notions to artificial intelligence and the cognitive sciences. Wheeler emphasizes the value of 'online intelligence' as contrasted to 'offline intelligence'. I discuss and apply these concepts with respect to worrying as it occurs in GAD, suggesting that GAD patients overrate the value of detached contemplation (offline intelligence), while underrating their embodied-embedded adaptive skills (online intelligence). I argue that this phenomenological account does not only help explaining why worrying is used as a coping strategy, but also why cognitive behavioral therapy is successful in treating GAD.

## 1. Introduction

Worrying is the core feature of generalized anxiety disorder (GAD) [1-4]. Many people worry from time to time, but in GAD the worrying is prolonged and difficult to control. Worrying is a way of coping with perceived threats. Yet, usually, it is not considered to be a helpful strategy for dealing with future situations [5], and the phenomenological account developed in this paper aims to show why.

There are various ways to explain the prolonged and uncontrollable worrying that occurs in GAD [2]. The present paper, however, is restricted to an explanation in phenomenological terms. Central to this account is to conceive of the human being as 'being-in-the-world' in the sense of an agent in continuous, embedded interaction with the environment. More specifically, my account is informed by Michael Wheeler's application of phenomenological (mainly Heideggerian) notions to artificial intelligence (AI) and the cognitive sciences. His approach - in which the concept of 'online intelligence' is central - enables us to articulate an unhelpful 'metacognition' in GAD (see next section), and, moreover, to explain why treating this condition requires cognitive as well as behavioral interventions. I suggest that the overall picture derived from this phenomenological perspective can also be communicated to GAD patients, hopefully providing them with additional motivation to find more helpful coping strategies than worrying.

The outline of the paper is as follows. First, I introduce GAD and the concept of worrying which is central in theorizing about GAD. Next, I present and discuss a phenomenological account of 'being in the world'. I start out explaining some central Heideggerian notions and then proceed to Wheeler's radical interpretation of these notions, articulated by the concept of 'online intelligence'. Then, I show how both accounts - theorizing about worrying in GAD on the one hand and online intelligence on the other - can be linked in order to explain why worrying in GAD occurs and why it is unhelpful. In brief, I suggest that in GAD there is a metacognition that overrates the value of detached contemplation about future situations while underrating the value and resources of actual embodied-embedded engagement.

## 2. GAD, worry, and metacognitions

GAD is an often chronic and impairing disorder with a lifetime prevalence estimated to be up to 5% in the United States population [4,6]. The core symptom is excessive or unreasonable worrying about all kinds of situations and the worrying is difficult to control [3,7]. There is a 15-20% heritability in GAD [6] and, in general, treatment options are cognitive behavioral therapy (CBT) [2,8] as well as medication [9].

Adrian Wells defines worrying as consisting of long chains of negative thoughts that are predominantly verbal in form and aimed at problem solving [5]. The common type of worrying - 'normal' worrying without GAD - is very similar in content to its GAD variant, but the GAD-type is less controllable [5]. Wells developed a model of worrying in GAD in which the concept of metacognitive beliefs and appraisals is central [5]. In this model, worrying is not just taken to be the natural consequence of being anxious. Instead, the model tries to explain *why *it is that the patient in an attempt to cope with fearsome stimuli *chooses *the worry-strategy above other coping options. Wells proposes that the reason behind the patient's choice is the presence of specific metacognitions about worrying. Patients, in other words, hold certain beliefs and appraisals about worrying that make them start and continue to worry in response to fearsome stimuli [10].

GAD patients do not worry all day; usually the worrying is triggered by thoughts with intrusive character, popping up in the patient's mind. They often take the form of "what if"-questions, like "what if I fail my exam?". Alternatively, they take the form of images of disasters [10]. It is at this point, confronted with such thoughts or images that metacognitive beliefs about the helpfulness of worrying come into play. Examples of such metacognitions are, as provided by Wells: "Worrying helps me cope; worrying keeps me safe; if I worry I'll be prepared." Such thoughts attach *positive value *to worrying. This hypothesis is supported by the observation by clinicians that GAD patients often maintain that worrying helps them to be prepared for negative outcomes [11]. In addition, GAD patients indeed turn out to believe that worrying is more helpful in finding solutions and preventing negative outcomes as compared to moderate worriers who do not fulfill GAD criteria [12]. GAD patients continue contemplating how to deal with possible scenarios until, at some point, they somehow feel that they will be able to cope. The assessment that the worrying process can be ended is, for example, based on internal cues like a "felt sense" that one is able to cope should the feared scenario unfold, or it may be related to some superstitious reasoning, or the worrying may stop because the patient is distracted [10].

Given the fact that the worrying is rooted in certain metacognitions (overarching ideas) attached to worrying, it is clear that in CBT GAD patients should learn to appreciate worrying differently. Wells discusses the case (and successful therapy) of a 25 year old and says the following about session 5 and 6 of this particular therapy: "Exposure to worried about situations was implemented (e.g., travelling alone, shopping alone) to test the accuracy of worry content against perceptions of real situations. This was intended to strengthen the replacement meta-belief that worries are inaccurate and therefore offer little advantage for coping." [5]

One element should be added to this account of metacognitions about worrying in GAD. As Riskind, referring to Beck, says ([2], p.2), individuals vulnerable to developing GAD "overestimate the magnitude and severity of threat, underestimate the extent of their coping resources, and overuse compensatory self-protective strategies such as cognitive, affective, or physical avoidance." [13] So, on such an account, GAD patients underestimate their actual coping skills with respect to real-life challenges [14]. In other words, in addition to the ideas about the value of *worrying *in coping with future situations, there is the underestimation of the patient's *own capabilities *for dealing with such situations should they occur.

It is characteristic for GAD patients not only to have positive ideas about worrying. In fact, according to Wells, these patients have negative thoughts about worrying as well. They worry about worrying itself, contemplating themes like: "I cannot control the worrying" and "worrying is harmful", "worrying means that I could go crazy," etcetera [5]. Wells calls these negative ideas about worrying 'type 2' worries. The worries about all other things in life are the - common - type 1 worries [5]. In this paper, I focus on type 1 worries, so the worries about future situations (and catastrophes).

We should note that in some patients the reason for worrying may be superstitious: Wells ([5], p. 303): "Such meta-cognitive beliefs may be linked to superstitious themes, such as not tempting fate through positive thinking, or to beliefs that worry is a good way of dealing with threat." In this paper, however, I will not go into the superstitious metacognitions, but concentrate on the non-superstitious metacognition that worrying somehow helps to prepare for future situations. In section 4, I propose that the metacognition that makes people choose for prolonged worrying as a coping strategy can be linked to a view of human agency that overrates detached contemplation and underrates embodied-embedded online intelligence in dealing with the world (see also next section).

## 3. Being-in-the-world and online intelligence

Phenomenologists, in particular the early Heidegger, suggest to conceive of the human agent^1 ^as being-in-the-world [15]. What is clear from this term by itself is that the world should not be conceived of as something separate from the human being, but as intimately related to what it is to be a human agent. Such a view stands in contrast with an observant, detached 'Cartesian Ego' that mainly contemplates about the world, as Michael Wheeler explains in more detail [16]. Furthermore, the phenomenological tradition conceives of the human being in *practical interaction *with the environment. According to Merleau-Ponty [17], "[c]onsciousness is in the first place not a matter of 'I think that' but of 'I can'". Notably, the 'I can' implies the possibility of *change *in the actual world. While the 'I think' refers to reflection about the world, the 'I can' acknowledges the continuous potential for change.

The phenomenological focus on interaction with the environment has resulted in specific terminology, emphasizing that worldly entities appear first of all as *tools*, as things that enable us to perform certain actions ([18], see also [19]). The objects are engaged in a way that has *already *appreciated the possibilities, options, or opportunities provided by these objects. Heidegger's famous example is his analysis of our appreciation of a hammer [15]. The hammer is not primarily perceived as a 'thing' with a wooden component and an iron part, but it has already been perceived - before its parts are recognized in their specific nature - as something which *enables *us to hammer. In everyday life, we engage the 'objects' as enabling certain actions, and therefore, indeed, as tools. Like when we are building, we pick up the tools without much thought; we are engaged in a certain praxis, which provides us with the practical eye that makes us recognize the specific tools suitable for the actions we intend to perform. In sum, we appreciate the world from a perspective that continuously recognizes actual possibilities for action.

The possibilities appreciated by the agent are not limited to one simple option [19]. Rather, we recognize a range or network of options. Returning to the hammer, this tool does not merely refer to the act of hammering; it rather opens up further possibilities, like fixing things and building a shed or a house. In fact, possibilities or options go on infinitely, because every possibility opens up a plethora of further possibilities [16]. In order to acknowledge that it is not about isolated possibilities, but about a range of interdependent options, Wheeler uses the term *involvement networks*: "...the hammer is involved in an act of hammering; that hammering is involved in making something fast; and that making something fast is involved in protecting the human against bad weather." [16] The analysis so far comes down to a basically action-oriented approach to our being in the world, in which practical options and possibilities provided by the *actual encounter *with the environment constitute the primary level of our understanding of the world.

Wheeler has integrated Heidegger's phenomenological analyses in the philosophy of artificial intelligence (AI) - in part building on Dreyfus's earlier work [20]. Wheeler is certainly not merely using *Heideggerian *phenomenological notions. A clear example is the central role of the body in Wheeler's account. In Heidegger's *Being and Time*, the body is absent, as Heidegger himself ([15], p. 143) says: "This 'bodily nature' hides a whole problematic of its own, though we shall not treat it here." Meanwhile, the body is to be found - and its role emphasized - in the work of *other *representatives of the phenomenological tradition, like Merleau-Ponty [17], who was one of the sources for Dreyfus's initial criticism of AI [21] (see also [16], p. 167) that inspired Wheeler's account. So, Wheeler's work is informed also by other phenomenological strands (other than Heidegger) enabling him to articulate the embodied nature of human agency.

According to Wheeler, engagement with the world is an *ongoing adaptive *process with continuous action-oriented perception [16]. He understands the engaged attitude toward the world as a form of 'online intelligence': "A creature displays online intelligence just when it produces a suite of fluid and flexible real-time adaptive responses to incoming sensory stimuli." This formulation shows a basic view of how both organisms (humans included) and robots relate to their environment - and is, indeed, a radical interpretation and application of some Heideggerian notions. Online intelligence is especially relevant in a world that is constantly changing, like our world. It is the opposite of offline intelligence: detached cognitive processes that are not in immediate interaction with the world, like contemplating the weather in Paris [16]. Offline intelligence, in other words, is the opposite of embodied-embedded cognitive activity. More can be said about such a distinction, and how it should be conceived of [22], but in this paper I intend to use it in Wheeler's sense.

For decades, Wheeler explains, people in AI tried to build robots equipped with cognitive maps of the world: these maps represented all kinds of aspects of the world. Contrary to what was expected, such robots were not able to *smoothly *interact with the environment. More recently, other types of robots emerged. These robots did not know that much about the world, but they were designed to continuously pick up environmental cues while interacting with the world. Without a precise representation of the world (so, without a map), but equipped with the capacity to continuously sense the world and interact with it, they are capable of smooth interaction. For this kind of robots their specific bodily features and abilities to interact are crucial [16]. So, without an elaborate map, but equipped with some relevant sensors, they are able to effectively interact with their environment - in a way their clumsy and detached predecessors were unable to.

In other words, an 'online' approach to the interaction with the world has been helpful to AI and robotics. It highlights that, contrary to what one might think, it is the *actual situation *that is the enabler of options and behavioral scenarios. In fact, AI for a long time overlooked the specific nature and complexity of interacting with the world. For example, three decades ago, IA specialists thought that the major challenge was building a chess computer able to beat the world champion. But beating a chess grandmaster - even the best of the (human) world - turned out to be the easy part. The real challenge was not this kind of cognitive activity, but real-time, online interaction with the world, like ants, mice, and falcons perform. Already several decades ago, Hubert Dreyfus identified and explained the problems and challenges in AI referring to Heideggerian notions like 'being-in-the-world' [16,20,23]. His account brought forward that interaction with the world is something much different from what we theoretically anticipated - still, in practice, we, as humans, are extremely good at it.

As indicated, the concept of online intelligence is intimately related to 'embodied-embedded cognitive science'. Wheeler ([16], p. 11) says, "[i]n its raw form, the embodied-embedded approach revolves around the thought that cognitive science needs to put cognition back in the brain, the brain back in the body, and the body back in the world." In my account I emphasize cognition, body, and the environment - not the brain. More in particular, I take the *situated *embodied nature of our being in the world to be the core of online intelligence within the context of this paper.

It is well established that the phenomenological tradition emphasizes the role of emotions in our being in the world [19,24,25]. This has to be acknowledged when considering profound disturbances of emotions - like in anxiety disorders, GAD being an example - from a phenomenological perspective. Heidegger, who also had a particular interest in psychiatry [26,27], explains that our engagement with the world is always taking place in some mood [15]. Mood, like the weather, is always there. We may be happy, sad, or anxious, but there is always a mood in which we engage our environment. And our mood is profoundly related to the actual options we appreciate in the environment [15,25]. It is, therefore, likely that anxious patients will appreciate and perceive all kinds of smaller and bigger threats in their environment because their mood makes them focus on such dangerous possibilities. So, an anxious patient won't have much trouble finding things to worry about and one could phenomenologically explore this issue with respect to GAD. However, this is not the focus of my paper; I take the 'online intelligence' angle on worrying in GAD.

Notably, usually online and offline cognitive activity go hand in hand: we combine these processes. In fact, in our everyday activities it is about a balance between these two, and about the ability to change one's approach in accordance with the task we perform.

In the next section I show how both accounts - concerning the unhelpful worrying strategy on the one hand and the online intelligence view on the other - can be linked in a way that brings forward a deeper metacognition in GAD: a metacognition about the nature of our interaction with the world.

## 4. Offline worrying versus online interaction

In the light of the analysis so far, how should we characterize worrying? It is a kind of thinking that takes a detached, offline - disembodied and disembedded - approach to future situations and scenarios that might become reality. Patients overusing such offline intelligence may be thinking all day about what it will be like to go to a supermarket and interact with the cashiers - whilst not actually going there but staying in bed instead. And without any of the environmental input that is in place in an actual supermarket, the patient can easily go on worrying for hours. The embodied approach recognizes and emphasizes that the human bodily resources are *richer *than is consciously available or accessible when detached contemplation is going on. The body is the means of online intelligence, especially equipped for actual engagement, not for offline contemplation about scenarios that may or may not become reality. In fact, it is virtually impossible to make a cognitive *map *for all events that may occur in a supermarket. Relying on such detached knowledge about the environment made it hard for robots to smoothly deal with their actual environment. Meanwhile, our online embodied cognitive capacities are perfectly capable of dealing with a vast variety of situations *at the moment they occur*. We are, as it appears, equipped for *actual *interaction rather than for detached contemplation about such interaction. This idea or 'metacognition' may be hard to accept for GAD patients, as it took time for AI scholars to accept its truth.

Let us take a further step. The metacognition with respect to the value of worrying on the part of the patient could be understood in the following terms: the patient appears to believe somehow that the offline, rational cognitive capacities can more or less be identified with our capacities as a whole. The idea of the human being is, in a way, equated with the rational Cartesian Ego: a detached mind, contemplating various scenarios. In contrast to such a Cartesian view of cognition and rationality stands the twentieth century pragmatic turn in phenomenology that focuses on actions (rather than thoughts), body, and a world or environment in flux (rather than the static object of detached contemplation). In other words, there is a parallel between the approach to being in the world that an exemplary GAD patient seems to take and a specific philosophical, Cartesian approach to the human agent. And since the GAD patients are likely to avoid the feared circumstances, the patients are (at least to some extent) deprived of the opportunity to *experience *the richness of their resources in actual online engagement. Consequently, their 'Cartesian' metacognition will remain uncorrected.

The gist of the analysis of the worrying process in terms of offline intelligence is that there appears to be a vital metacognition, namely that offline contemplation is the means of choice for preparing for future situations - ignoring the online cognitive and behavioral resources. We also noted that GAD patients tend to underestimate their ability to deal with whatever scenarios might obtain in reality. Taking these together, I propose the following perspective on worrying in GAD: These patients may underestimate their coping skills in such feared situations, because they tend to largely overlook their embodied-embedded resources, while they are convinced that detached contemplation is a helpful means to deal with the world. In fact, overlooking the online aspect of the human nature cannot but result in an underestimation of the actual skills one possesses.

Although the focus of the present paper is on metacognitions, it is important to note that GAD is not just a cognitive stance on our interaction with the environment, but a disorder in which people are anxious, and, therefore, a disorder that is about mood, emotions, stress and distress. The burden to the patient will often consist of - apart from avoidance - continuous anxiety and stress. Still, in this paper I focus on unhelpful (meta)cognitions, not on emotional distress.

It is clear that offline contemplation is not at all wrong or bad in itself; it is merely the uncontrolled and prolonged contemplation as it occurs in GAD that is unproductive and unhelpful. In fact, since we are no Cartesian Ego's but embodied and embedded agents - not going anywhere without our bodies - the contemplation-strategy should be used *selectively*. We will never be able to cognitively grasp completely how we do what we do every day. Many of our own online capacities are likely to remain a mystery to ourselves: we do not even know how we raise our own arm. Thinking about behavioral responses will never be the same as actual engagement, and is, therefore, indeed, only of limited value.

The worry-strategy is not only unproductive; it also stimulates avoidance of the actual situations. I mentioned that the avoidance of feared situations indeed deprives the patient of the opportunity to *correct *the metacognition by showing that the patient can actually deal with the situation. The (repeated) behavioral experience that one is able to deal with actual situations is probably the strongest means to achieve metacognitive correction. This could also be part of the explanation of why it is that the behavioral element of CBT is successful in treating GAD [28]. In section 2, the case of a 25 year old was mentioned and session 5 and 6 of CBT contained a behavioral component: "Exposure to worried about situations was implemented (e.g., travelling alone, shopping alone) to test the accuracy of worry content against perceptions of real situations. This was intended to strengthen the replacement meta-belief that worries are inaccurate and therefore offer little advantage for coping." [5] Such a behavioral approach is completely understandable from the phenomenological perspective proposed in this paper. Online intelligence should be *experienced *and *performed *as a means to achieve correction of the metacognitions.

A specific behavioral aspect of CBT in anxiety disorders in general could also be explained from an embodied-embedded perspective. Anxiety patients may be afraid that they will be overwhelmed by anxiety in the feared or worried about situations. And these patients are right (at least in part): in such feared situations anxiety levels will increase, and usually these patients will flee from the situation as soon as possible. Yet, in CBT, anxiety patients are motivated to expose themselves to such stressful situations *without *fleeing. In such cases anxiety levels will initially increase - but since patients are not fleeing from the stressor, they will experience that the (extreme) anxiety levels eventually drop (often they decrease after, let's say, 10-20 minutes). Thus, CBT enables the patients to experience their bodily responses and to find out that, after a period of time, anxiety levels actually *decrease *in the feared situation. There is ample evidence for the effectiveness of this exposure component of CBT [29]. On the phenomenological account developed so far, this is just one example of our adaptive, online bodily capacities and responses. Our body *adapts *- physiological anxiety responses decrease - to a situation in a way a worrying patient might well overlook. In fact, our bodily responses, like the physical responses to stressors, are not static, but dynamic in nature, and aimed at effective coping. The behavioral component of CBT, therefore, shows the patients their own online responses to the feared situation.

Phenomenologically informed research has provided evidence that 'disembodiment' is a problem in several psychiatric disorders, like schizophrenia and melancholia [30,31]. In such cases of a 'disembodied mind' there is a bodily (mediated) disconnect between the mind and the environment. For instance, in melancholia patients may feel completely alienated from the world, not 'in touch' with it anymore. Still, there is a difference between this kind of 'disembodiment' and the approach to GAD developed in the present paper. For this paper is not about actual disembodiment or perceptual changes in GAD, but about *metacognitions *on the part of the patient *pertaining to *his interaction with the environment. In other words, in (more severe) disorders like schizophrenia, there are actual (developmental) changes in bodily interaction with the world, whilst in GAD the main problem concerns metacognitions about how to deal with future situations. Meanwhile, it would be interesting to study GAD in the way that has been done in melancholia and schizophrenia[32], aiming to find signs of actual, e.g., perceptual disembodiment.

The idea put forward in this paper could also be explained to patients, I suggest. In brief, a therapist could say something like this to the patient: "You are more resourceful than you think you are, because you focus and rely on detached contemplation skills. In fact, however, you are ignoring that you are a human being, which means a being that has the specific ability to deal with real life situations rather than with contemplated ones. Granted, detached deliberation and contemplation are indispensible features of our being in the world. Yet, their value is limited; as soon as contemplation takes over and becomes uncontrollable - becomes worrying -, your cognitive endeavors stop adding to your resources in real life situations. Part of the therapy will be to experience your own behavioral resources in the situations you fear. We will do this gradually, but over time you will be surprised by your own capacities in dealing with *actual *situations." Explaining this view to patients might help motivating them to, indeed, engage bodily - instead of via worrisome imagination - in (feared) situations, and experience their own adaptive skills. In addition, providing patients with this overall picture might encourage them to identify specific aspects of their own behavioral responses in the feared situation that were unanticipated, i.e., overlooked in the worry-scenarios - yet were effective in dealing with the stressor. Identifying such elements of their own behavior could deepen their belief that worrying is not a helpful strategy.

Still, the emphasis on correcting the metacognition about how to deal with the world, should not suggest that other metacognitive corrections are irrelevant when treating GAD. Usually, in CBT, the therapist and the patient identify various (meta)cognitions that contribute to the GAD symptoms in that particular patient. Identifying and correcting these specific (meta)cognitions is a powerful part of CBT. Meanwhile, I suggest that there appears to be an overarching or deep metacognition which ignores the resources of online intelligence. Correcting this metacognition could be part of CBT as well.

## 5. Conclusions

GAD patients hold certain metacognitions, circling around the value of worrying as a way of preparing for future events. Such metacognitions are shared by many people, but in GAD they are more extreme. In addition, GAD patients tend to underrate their own coping skills should the feared situation occur. I related these phenomena to a philosophical - 'Cartesian' - position that concentrates on rational deliberation, while (at least in part) ignoring our embodied-embedded existence. More specifically, from the perspective of an online account, the GAD-metacognitions are in fact mistakes about what we are. Like philosophers tended to ignore the body as well as the environment (embeddedness), GAD patients tend to either ignore or underrate the embodied-embedded skills human agents naturally have. From this biased perspective it becomes understandable that people start figuring out all kinds of scenarios in a detached offline fashion and at the same time tend to avoid the actual situation just as long as they do not feel that they have worked out all sorts of eventualities. The online intelligence approach, however, acknowledges that, indeed, the future is uncertain (the world being in flux). Meanwhile, at some point detached contemplation adds very little to our coping resources; its value is limited.

The proposed view adds to our understanding of why CBT is effective in these patients: on the one hand it aims at direct *cognitive *correction of the relevant GAD-metacognitions; on the other hand its *behavioral *component provides the GAD patients with the experience that their resources are much richer than prolonged (fearful) offline contemplation can imagine.

Since many people value worrying to some extent and many people do it from time to time, it might be that more people do not fully realize the limitations of offline contemplation on the one hand and the resources of online intelligence in dealing with our world on the other.

## List of abbreviations

AI: Artificial intelligence; CBT: Cognitive behavioral therapy; GAD: Generalized anxiety disorder.

## Competing interests

The author declares that he has no competing interests.

## Authors' contributions

GM is responsible for the full content of the manuscript.

## Author's information

Gerben Meynen received a PhD in Philosophy and in Medicine. In 2007 he started his current research project (NWO-Veni grant) on free will and mental disorder at the Faculty of Philosophy, VU University Amsterdam. He also works as a psychiatrist at the Outpatient Clinic for Anxiety Disorders at GGZ InGeest, partner of the VU Medical Center, Amsterdam. His main research interest is philosophy and ethics of psychiatry.
